# Global and regional DNA methylation patterns in heart failure: a case–control analysis

**DOI:** 10.1016/j.ebiom.2026.106340

**Published:** 2026-06-23

**Authors:** Mykhailo Krolevets, Vincent ten Cate, Jürgen H. Prochaska, Andreas Schulz, Steffen Rapp, Silav Zeid, Stefan Tenzer, Miguel A. Andrade-Navarro, Ake Lu, Konstantin Strauch, Alexander K. Schuster, Manfred E. Beutel, Isabel Heinrich, Julia Weinmann-Menke, Karl J. Lackner, Philipp Lurz, Steve Horvath, Christof Niehrs, Philipp S. Wild

**Affiliations:** a Preventive Cardiology and Preventive Medicine, Department of Cardiology, University Medical Center of the Johannes Gutenberg University, Langenbeckstr. 1, Mainz 55131, Germany; b Institute of Molecular Biology (IMB), Mainz 55128, Germany; c Systems Medicine, Institute of Molecular Biology (IMB), Ackermannweg 4, Mainz 55128, Germany; d German Center for Cardiovascular Research (DZHK), Partner Site Rhine Main, University Medical Center of the Johannes Gutenberg University Mainz, Mainz, Germany; e Clinical Epidemiology and Systems Medicine, Center for Thrombosis and Hemostasis (CTH), Mainz, Germany; f Institute for Immunology, University Medical Center of the Johannes Gutenberg-University Mainz, Mainz, Germany; g Institute of Organismic and Molecular Evolution, Johannes Gutenberg University Mainz, Mainz, Germany; h Altos Labs, San Diego, USA; i Institute of Medical Biostatistics, Epidemiology and Informatics, University Medical Center of the Johannes Gutenberg-University Mainz, Mainz, Germany; j Department of Ophthalmology, University Medical Center of the Johannes Gutenberg University Mainz, Mainz, Germany; k Department of Psychosomatic Medicine and Psychotherapy, University Medical Center of the Johannes Gutenberg University Mainz, Mainz, Germany; l Department of Psychiatry and Psychotherapy, University Medical Center of the Johannes Gutenberg University Mainz, Mainz, Germany; m Department of Nephrology and Rheumatology, Center of Immunotherapy, Medical Center of the Johannes Gutenberg University, Mainz, Germany; n Institute for Clinical Chemistry and Laboratory Medicine, University Medical Center of the Johannes Gutenberg University Mainz, Germany; o Department of Cardiology, University Medical Center of the Johannes Gutenberg University, Langenbeckstr. 1, Mainz 55131, Germany; p Department of Medicine, David Geffen School of Medicine, University of California, Los Angeles, USA; q Division of Molecular Embryology, DKFZ-ZMBH Alliance, Heidelberg 69120, Germany; r Department of Nephrology, Heidelberg University Hospital, Im Neuenheimer Feld 162, Heidelberg 69120, Germany

**Keywords:** Heart failure, Epigenetics, DNA methylation, Cardiovascular, Global methylation

## Abstract

**Background:**

Heart failure (HF) is a syndrome of heterogeneous aetiology and multiple exposomal causes. The role of DNA methylation (DNAm) changes in symptomatic HF and its phenotypes is unclear. This work addresses global and regional DNAm patterns and epigenetic ageing in HF.

**Methods:**

Patients with HF were included from the MyoVasc Study, an investigator-initiated prospective cohort study. Individuals without HF were included from a population-based Gutenberg Health Study. DNAm was measured using the Illumina Infinium MethylationEPIC assay. Epigenetic ageing was calculated using GrimAge and Hannum methylation clocks.

**Findings:**

Global DNAm patterns were analysed across 767,735 CpG sites in a dataset of N = 2155 individuals. HF was associated with an increase of epigenetic age by on average 19.0 [11.0; 29.0] years and with a global decrease in methylation across all phenotypes (Odds ratio [OR]: 1.33 [95% CI: 1.17–1.51], p < 0.0001). Epigenetic clocks strongly predicted incident worsening of HF and mortality. Methylation differences varied across chromosomes and were most pronounced in shore (OR: 1.41 [95% CI: 1.25–1.61], p < 0.0001) region. CpG island methylation had a weaker association with HF status (p = 0.0122), but strongly interacted with methylation in adjacent regions.

**Interpretation:**

This work highlights accelerated ageing, global and regional methylation patterns as key features associated with the presence, severity, and consequences of HF. HF-related methylation exhibited diverse patterns across chromosomes, the genome, and gene regions. CpG islands lacked strong discriminatory capacity, but their potential regulatory role is a novel finding.

**Funding:**

SHARP, BMBF, DZHK, CTVB, DIASyM, Stiftung Rheinland-Pfalz für Innovation, ReALity, curATime.

**Research in context**

**Evidence before this study**

We searched PubMed and Web of Science for English-language publications up to November 2025 using the query (“DNA Methylation” OR “Global Methylation”) AND (“Heart Failure” OR “HF”). Heart failure syndrome is heavily driven by environmental exposures with many lifestyle factors playing a substantially bigger role, compared to individual genetic background. At the same time, an epigenetic modification of the DNA strand—DNA methylation is known to reflect environmental exposures over long periods of time. One of the ways of aggregating this information from the whole genome to a single value per individual is global methylation—average across all the methylated positions. To date, however, there is very limited knowledge on the association between global methylation and heart failure.

**Added value of this study**

We have analysed DNA methylation patterns and clinical data from 2155 individuals with and without heart failure from two prospective cohorts, calculating their global DNA methylation as well as multiple epigenetic clocks. We found that the degree of demethylation was related to the severity of the syndrome and epigenetic clocks were predictive for clinical outcome as reflected by the endpoints worsening of HF and mortality. The difference in global methylation between heart failure and heart failure-free corresponded to around 19 years of age. Patterns of HF-related methylation changes varied across chromosomes, genome and gene regions and were most pronounced in shore regions and upstream of the transcription start sites. Despite a low discriminatory power, these findings suggested potential regulatory role of CpG islands for methylation in adjacent regions.

**Implications of all the available evidence**

Taken together, this data suggests that global DNA methylation can server as a cumulative marker of presence and severity of HF as well as provide deeper insights into its biology. While further investigations are required to fully understand the role of DNA methylation in heart failure, these results provide a strong foundation for further research.

## Introduction

The most frequent target of DNA methylation in mammals is a CpG dinucleotide. DNA methylation can silence or promote gene expression, depending on the methylation of a specific position or a whole genome region^1^ and is influenced by a plethora of environmental factors, including diet, physical activity, stress, tobacco consumption and exposure to airborne pollutants.^2^ Hence, the methylome can serve as a biological readout for the cumulative environmental factors to which an individual was exposed.

Heart failure (HF) is a condition that affects millions of individuals worldwide in 2024^3^ and relates to a relevant economic burden.^4^^,^^5^ HF is associated with poor prognosis and accounted for more than 425,000 deaths and for 45% of cardiovascular deaths in the U.S. in 2021 alone.^6^ The syndrome is primarily an environmentally driven condition, with lifestyle factors^7^ including diet, physical activity and stress^8^ contributing substantially more risk than genetic predisposition.^9^ The 10-year incidence for offspring of individuals with HF is 2.7% compared to 1.6% for those without family history.^10^

Several studies have explored links between HF and DNA methylation,^11^, ^12^, ^13^, ^14^, ^15^ discovering individual methylation sites associated with HF, with some focusing on phenotypes of HF.^16^^,^^17^ However, no studies to date have investigated global and regional DNA methylation, e.g., by gene region, genome regions or by chromosome. Furthermore, differences in global DNA methylation between HF phenotypes as well as the relationship with epigenetic ageing remain unexplored.

This study aimed to address these gaps in our understanding of epigenetic modifications that arise with the development and progression of heart failure.

## Methods

### Sample for investigation

For the investigation, individuals from two large cohorts from the same geographical region of Germany were included in a case-control setting. Patients with heart failure were included from the MyoVasc Study (NCT04064450; N = 3289)—a large investigator-initiated, prospective cohort study on HF located at the University Medical Centre Mainz in Mainz, Germany, that was conducted through January 2013 to April 2018. Information on the rationale and design of the cohort, including information on all study procedures has been reported in detail.^18^ For the present analysis, individuals from the cohort with presence of symptomatic HF (i.e., American College of Cardiology Foundation/American Heart Association Stage C/D), but no pregnancy, presence of an intracardiac implant or history of heart transplant, were included.

Individuals without HF were included from the Gutenberg Health Study (GHS) as control group. GHS is a large population-based, prospective, single-centre cohort study conducted in Mid-Western Germany between April 2007 and April 2017 (constituting the first survey, N = 15,010). Study design and detailed information on the GHS have been published.^19^ Individuals without cardiovascular disease (including HF, atrial fibrillation, history of venous thromboembolism, history of myocardial infarction, history of stroke, coronary artery disease), cancer, chronic obstructive pulmonary disease, autoimmune disease, acute infection, chronic kidney disease (i.e., eGFR ≤60 ml/min/1.73 m^2^), or active pregnancy, were included in the control sample. Only participants with a documented visit in the study centre at both 5 and 10 years of follow up were included in this analysis to assess subsequent health trajectories. Control subjects were selected from this pool to achieve the closest possible alignment in age and sex distributions with the HF cases, as exact matching was not feasible.

The MyoVasc and GHS cohorts were both recruited from the same geographical region in Midwest Germany, ensuring a highly comparable environmental and genetic background. To minimise technical confounding, identical protocols were strictly followed for both cohorts.

### Ethics

The study conformed to the principles outlined in the declaration of Helsinki and was approved by ethics committees (reference numbers: GHS: 2018-13720, MyoVasc: 837.319.12 (8420-F)). All participants provided written informed consent before study inclusion.

### Definition of heart failure phenotypes and clinical endpoints

Stages of HF were defined in accordance to the Universal Definition and Classification of Heart Failure. Accordingly, three HF phenotypes from stages C and D were defined based on LVEF and diastolic dysfunction: heart failure with preserved ejection fraction—HFpEF (LVEF ≥ 50%, present diastolic dysfunction), heart failure with mildly reduced ejection fraction HFmrEF (LVEF > 40% to <50%) and heart failure with reduced ejection fraction—HFrEF (LVEF ≤ 40%). The clinical outcome of primary interest was defined as a worsening of HF, as a composite of the endpoints cardiac death and hospitalisation for HF. Diagnoses were obtained from medical reports and self-reported data and assessed by a board-certified cardiologist. All-cause death was determined via annual computer-assisted structured telephone interview and quarterly checks by the German registration offices.

### DNA isolation and methylation measurements

Within 2 h after blood drawing, DNA was extracted from peripheral blood using salting, dissolved in TE buffer and stored at −80 °C in the Biomaterialbank, Mainz, a state of the art biobanking facility. DNA methylation measurements were performed using the Infinium MethylationEPIC BeadChip platform that encompasses 866,554 individual CpG methylation positions (Illumina, San Diego, CA, USA). The arrays were processed according to the HD Methylation protocol by Illumina and converted using the EZ-96 DNA Methylation-Lightning MagPrep kit (Zymo Research Corporation, Irvine, CA) according to manufacturer instructions. The resulting IDAT files represent summary light intensities for each probe-type in an array.

To minimise potential batch effects and ensure data consistency, all blood samples from both cohorts were collected, processed, and analysed using a strictly uniform protocol. Blood withdrawal, sample selection, and DNA extraction were performed by the same team of technicians. All methylation measurements were conducted on the same equipment and within the same laboratory facility. Furthermore, the resulting array data were processed and normalised jointly for both cohorts to ensure identical handling of raw signals. This integrated approach was designed to mitigate systemic technical variation, and subsequent statistical checks were performed to confirm that any residual batch effects were minimised.

### Methylation data preprocessing

The raw IDAT files were loaded and processed using the statistical programming environment R version 4.1.0 with the use of the ChAMP package, which resulted in the bead intensity values being converted to beta-values (a proportion of cells in which a given site is methylated) in the range from 0 to 1 for each individual CpG site. Next, all CpG sites with a detection p-value > 0.01 were removed, followed by filtering of all CpG sites with a bead count <3 in at least 5% of the samples. Both sex chromosomes were excluded from this analysis due to potential dosage imbalance and X-chromosome inactivation. Subsequently, the filtered data were corrected for the effect of type-I and type-II probes. The MethylationEPIC array uses Infinium I (type I) and Infinium II (type II) probes to increase genomic coverage. However, the different composition of probes results in different type I and II methylation value distributions when using BMIQ normalisation. Singular value decomposition (SVD) was performed to determine which technical confounders were related with the principal component scores of methylation beta values (FDR-adjusted two-sided p < 0.05). Batch effect correction was performed using the ComBat method. Global methylation was calculated across the whole genome, chromosomes, and gene and genome regions (as defined in the Infinium MethylationEPIC v1.0 Manifest file; gene regions include: intergenic region, transcription start site 1,500, transcription start site 200, 5'UTR, 1st Exon, Exon boundary, gene body and 3'UTR; genome regions included: genome regions refer to genomic regions in relation to nearest CpG island: island, shore—2000 base pairs away, shelf—4000 base pairs away, open sea—everything else), by taking the average across all respective CpG sites for the given sample, i.e., $\frac{1}{p_{j}}\sum_{i=1}^{p_{j}}\beta_{ijk}$, where p_j_ denotes the number of CpG sites in the jth region type (e.g. whole genome, chromosome, gene region), and $\beta_{ijk}$ the ith methylation beta value in the jth region for the kth individual.

### Relationship between cardiovascular risk factors and global methylation

The relation between traditional cardiovascular risk factors (age, sex, active smoking, arterial hypertension, diabetes mellitus, dyslipidemia, obesity and family history of myocardial infarction or stroke) and global methylation was determined using linear regression with and without adjustment for age and sex (age was adjusted only for sex and sex was adjusted only for age). All further models were adjusted for age and sex.

### Statistical analysis

All statistical analyses were performed using the statistical programming environment R, version 4.1.0. The difference in global methylation between groups was tested using linear model with and without adjustment for age. The relationship between age and global methylation, as well as premature ageing and severity of HF was modelled using linear regression. Interaction analysis was performed using a linear regression model including age as an interaction term.

Relationship of age and global methylation for HFpEF, HFmrEF and HFrEF was modelled with adjustment for NT-proBNP.

To quantify the biological ageing associated with HF, a “methylation age gap” was estimated using a bootstrapping approach. For each of 10,000 bootstrap iterations, a linear regression model was fitted to the data according to the following formula:

$$Global\;methylation=\beta_{0}+\beta_{1}Age+\beta_{2}HF\;Status+\varepsilon$$

The inclusion of HF status as a covariate allows the model to isolate the independent effect of the disease on methylation levels while holding chronological age constant. Following the model fit, the chronological age required to reach a specific methylation threshold was calculated for both the HF and control groups. The “methylation age gap” was then defined as the difference in years between these two calculated ages. This approach effectively translates the vertical displacement between the two regression lines ($\beta_{2})$ into a horizontal unit of time (years). Statistical significance and 95% confidence intervals for this difference were derived from the 2.5th and 97.5th percentiles of the resulting bootstrap distribution. To account for the confounding effect of varying cell types, cell-type proportions (CD8+ T-cells, CD4+ T-cells, Natural Killer cells, B-cells, Monocytes, and Granulocytes) were estimated from the DNA methylation data using the estimateCellCounts function of the ‘minfi’ and ‘FlowSorted.Blood.450k’ R packages.

Epigenetic clocks Hannum clock and GrimAge were generated according to methods described in their respective publication.^20^^,^^21^ The Hannum clock was selected as a well-validated first-generation estimator of systemic biological ageing. GrimAge, a second-generation clock trained on mortality and plasma protein surrogates, was prioritised for its superior performance in predicting lifespan-related outcomes and age-related clinical decline. Premature ageing was operationalised as residuals from linear regression models predicting epigenetic age according to an epigenetic clock with calendar age. Higher premature ageing scores indicated higher epigenetic age when compared with calendar age. Logistic regression models with robust variance estimators adjusted for age and sex were used to generate odds ratios, confidence intervals and p-values for HF phenotypes and regional methylation analysis. Worsening of HF and total mortality in individuals with HF were modelled by Cox regression with adjustment for age and sex. To test whether the variability of methylation difference between HF and control differed by chromosomes, an asymptotic test for the equality of coefficients of variation was used, where the null hypothesis indicated an absence of difference. Modulation of the relationship between region-specific methylation and HF by methylation in other regions was modelled using individual regression models with interaction terms between region-specific global methylation variables.

To evaluate potential residual confounding by age and cohort structure, age distribution overlap between HF cases and controls was quantified using density overlap coefficients defined as the integral of the minimum of the two kernel density estimates. To further assess robustness, repeated age matching was performed. In 1000 iterations, 50% of HF cases were randomly subsampled and matched to controls on age using nearest-neighbour matching with a calliper of 0.2. Post-match age differences and distributional overlap coefficients were calculated for each iteration. Group differences in global methylation were re-estimated across all matched samples to assess stability of the findings.

### Role of the funding source

The funders had no role in study design, interpretation, data collection, analysis, writing of the manuscript or the decision to submit the paper for publication.

## Results

### Sample characteristics and distribution of global methylation

Whole blood samples from 2161 study participants were subjected to DNA methylation measurement (cases: n = 1317 individuals with objectively diagnosed chronic heart failure, referred to as HF individuals; controls: n = 838 individuals without clinically confirmed heart failure, referred to as control individuals). In total, 99.9% of samples were successfully analysed with sufficient quality and concentration of DNA. Additionally, three samples were excluded after preprocessing due to poor signal detection quality (p-value > 0.01) reported by the measuring device. Out of 866,554 individual methylated positions measured on the Infinium Methylation EPIC assay, 767,735 remained after filtering. The sample plate, sample well, slide, array, scanner, date and hour of measurement were identified as technical confounders that correlated with methylation levels (Supplemental Fig. S1); batch effect correction by ComBat accounted for these factors (Supplemental Fig. S2). Global DNA methylation was assessed in 2155 individuals (1317 HF and 838 control individuals). Of these individuals, 38.6% (HF: 32.0%; controls: 49.0%) were female. Mean age was 62.8 ± 11.8 years (HF group: 68.4 ± 9.7; control group: 54.1 ± 9.4).

HF was stratified into three phenotypes based on LVEF: HFpEF (HF with preserved EF), defined as LVEF ≥50% (N = 605); HFmrEF (HF with mildly reduced EF), with LVEF 41–49% (N = 383); and HFrEF (HF with reduced EF), where LVEF is ≤ 40% (N = 329). Details are presented in Supplemental Table S1. The global level of genome methylation was lower in HF than in control subjects (0.609 ± 0.004 vs. 0.611 ± 0.003, p = 2.56 × 10^−37^, p adjusted for age = 6.6 × 10^−9^; (Supplemental Fig. S3A and B). Although the absolute difference in global methylation was small, it reflects a consistent shift across more than 760,000 CpG sites, indicating a system-wide epigenomic alteration rather than a locus-specific effect. Adjusting for age, sex and leucocyte composition (CD8+ T, CD4+ T, NK, B-cells, Monocytes, and Granulocytes; Supplemental Fig. S4) attenuated the effect, yet the association between global methylation and HF remained (β (SD): -0.41, 95% [confidence interval (CI): −0.52 to 0.29], p = 3.04 × 10^−11^). The association between global DNA methylation and HF status remained after adjusting for age, sex, smoking, alcohol consumption, β-blockers, and ACE inhibitors (β = −0.24, 95% CI: −0.41 to 0.07, p = 0.006).

The baseline age distribution showed overlap between HF cases and controls of 0.48; (Supplemental Fig. S5A). After repeated propensity score matching, median post-match age differences were reduced to 0.69 years compared with 14.3 years in the full sample, with substantial improvement in distributional overlap (median overlap coefficient = 0.93; (Supplemental Fig. S5B and C). Across 1000 iterations, the direction of the effect (lower global methylation in HF) remained consistent between groups (Supplemental Fig. S5D and E). The median global methylation difference across iterations was −0.0012 compared to unmatched analysis of −0.0021.

### Relation between global methylation, age and cardiovascular risk factors

**Fig. 1:**
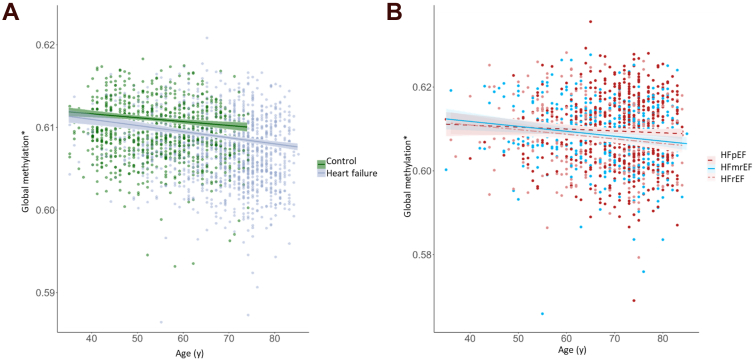
Heart failure and global methylation. A—Global methylation in HF and controls. B—Global demethylation in relationship with age and disease phenotype. Methylation is defined as a percentage of methylated CpG on a given position in Genome. i.e., 0 = 100% are demethylated, 1 = 100% are methylated. ∗Global methylation is defined as average across all available CpG for given sample. Linear regression lines are generated with age as predictor and global methylation as dependent variable. CI—confidence interval; HFpEF–HF with preserved ejection fraction; HFrEF–HF with reduced ejection fraction; HFmrEF–HF with mildly reduced ejection fraction. HFpEF, HFmrEF and HFrEF curves are adjusted for NT-proBNP.

On average, the global methylation level observed in individuals with HF corresponded to the level typically seen in control subjects who were 19.0 years older [95% bootstrap CI: 11.0–29.0] (Fig. 1A). Global methylation showed a negative association with age in both the HF group (p = 7.83 × 10^−11^) and the HF-free (p = 0.0002) group. No interaction between group status and age was observed (p = 0.125). Similar patterns were observed across HF phenotypes where global methylation levels matched those of controls with increased age (HFpEF: 20.0 years [95% CI: 8.0; 30.0]; HFmrEF: 20.8 years [95% CI: 11.0; 29.0]; HFrEF: 24.6 years [95% CI: 15.0; 30.0]) (Fig. 1B). These differences were independent of the severity of HF, as the effect of NT-pro-BNP was removed from the methylation difference.

The relationship between traditional cardiovascular risk factors and global methylation, with and without adjustment for age and sex, is shown in Table 1. Age, arterial hypertension, diabetes mellitus, dyslipidemia, and obesity were negatively associated with global methylation in crude models while female sex was positively associated. After adjusting the models for age and sex (age was only adjusted for sex and vice versa), all relationships except those of global methylation with age and sex were lost.

**Table 1:** Assessment of association of traditional cardiovascular risk factors with global methylation.

	Unadjusted			aSex and age adjusted		
	N = 2155			N = 2155		
	β-estimate [95% CI]	p-value	p for interaction with HF	β-estimate [95% CI]	p-value	p for interaction with HF
Age [SD]	−0.11 [−0.13; −0.09]	<0.0001	0.12	−0.10 [−0.12; −0.09]	<0.0001	0.04
Female sex	0.09 [0.07; 0.10]	<0.0001	0.11	0.08 [0.06; 0.09]	<0.0001	0.04
Active smoking	0.01 [-0.01; 0.03]	0.33	0.15	−0.01 [−0.03; 0.01]	0.24	0.17
Arterial hypertension	−0.06 [−0.08; −0.04]	<0.0001	0.73	−0.01 [−0.03; 0.01]	0.23	0.73
Diabetes mellitus	−0.05 [−0.06; −0.03]	<0.0001	0.40	−0.01 [−0.03; 0.01]	0.18	0.16
Dyslipidemia	−0.06 [−0.08; −0.05]	<0.0001	0.16	−0.01 [−0.03; 0.01]	0.23	0.47
FHx MI or stroke	0.005 [-0.01; 0.02]	0.53	0.73	0.03 [0.01; 0.05]	0.87	0.27
Obesity	−0.02 [−0.04; −0.01]	0.007	0.11	−0.01 [−0.03; 0.01]	0.11	0.47

Estimates and p-values as well as p-values of interaction with heart failure status generated with linear regression models. Each row is a separate model. SD—standard deviation; FHx—family history; MI—myocardial infarction; CI—confidence interval.

a Age is adjusted only for sex and sex is only adjusted for age.

### Relation between premature ageing with heart failure severity and outcome

**Fig. 2:**
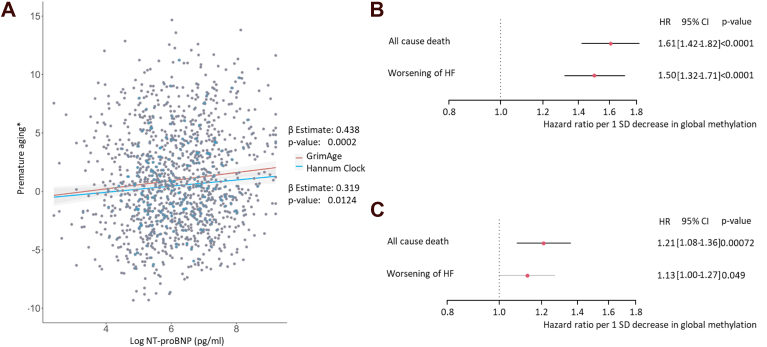
Relation of premature ageing with HF severity and clinical outcome. A—Premature ageing in relation to HF severity. B—Impact of premature ageing derived from GrimAge on clinical outcome. C—Impact of premature ageing derived from Hannum Clock on clinical outcome. ∗Premature ageing—residuals from a linear regression of calendar age predicting epigenetic age. The residuals represent the portion of epigenetic age not explained by calendar age, where positive residuals indicate accelerated ageing with respect to one’s calendar age. NT-pro-BNP–natriuretic peptide level. Premature ageing was derived from Hannum Clock and GrimAge.^20^^,^^21^ Accelerated ageing and worsening of HF generated using Cox regression. HR—hazard ratio. CI—confidence interval. Models adjusted for age and sex.

Premature ageing, as derived from epigenetic clocks such as the Hannum Clock^20^ and GrimAge,^21^ was positively associated with the severity of heart failure, defined on the basis of NT-pro-BNP, a protein with highly cardiac tissue-specific expression and the clinical gold standard laboratory marker for HF severity (Fig. 2A). Premature ageing was predicting all-cause death and worsening of heart failure for both epigenetic clocks in a follow-up period of 10 years (all-cause death mean time-to event = 3.6 ± 2.0; worsening of heart failure = 3.3 ± 2.0) (Fig. 2B and C).

To determine the robustness of the epigenetic ageing signals, sensitivity analyses were performed with adjustment for estimated cell-type proportions. Accelerated ageing derived from GrimAge remained predictive of both all-cause mortality (HR: 1.56, 95% CI: 1.38–1.76, p < 0.0001) and worsening of HF (HR: 1.40, 95% CI: 1.24–1.60, p < 0.0001) after accounting for cell-type composition (Supplemental Fig. S6A). In contrast, associations of Hannum clock-derived accelerated ageing with both outcomes were attenuated after accounting for cell-type composition (mortality: HR: 1.12 [95% CI: 1.00–1.26], p = 0.06; worsening of HF: HR: 1.04 [95% CI: 0.92–1.18], p = 0.51; Supplemental Fig. S6B). Similarly, the prognostic value of accelerated ageing derived from GrimAge remained robust in models adjusted for age, sex, smoking, alcohol intake, β-blockers intake and ACE inhibitors intake for both all-cause mortality (HR: 1.66 [95% CI: 1.46–1.88], p < 0.0001) and worsening of HF (HR: 1.53 [95% CI: 1.34–1.75], p < 0.0001; Supplemental Fig. S7A). For the Hannum clock, the effect on mortality remained (HR: 1.20 [95% CI: 1.07–1.34], p = 0.0015), but the effect on worsening of HF diminished (HR: 1.11 [95% CI: 0.99–1.25], p = 0.074; Supplemental Fig. S7B).

The value of accelerated ageing derived from GrimAge remained prognostic after adjusting for eight classical cardiovascular risk factors (age, sex diabetes type 2 mellitus, obesity, smoking status, arterial hypertension, dyslipidemia and family history of myocardial infarction or stroke) for both all-cause mortality (HR: 1.65 [95% CI: 1.46–1.88], p < 0.0001) and worsening of HF (HR: 1.48 [95% CI: 1.29–1.69], p < 0.0001; Supplemental Fig. S8A). Mortality remained associated with Hannum clock-derived accelerated ageing after adjustment (HR: 1.18 [95% CI: 1.06–1.32], p = 0.0031), but the association with worsening of HF was attenuated (HR: 1.09 [95% CI: 0.97–1.23], p = 0.14; Supplemental Fig. S8A).

### Global methylation according to phenotypes of heart failure and its relation to clinical outcome

**Fig. 3:**
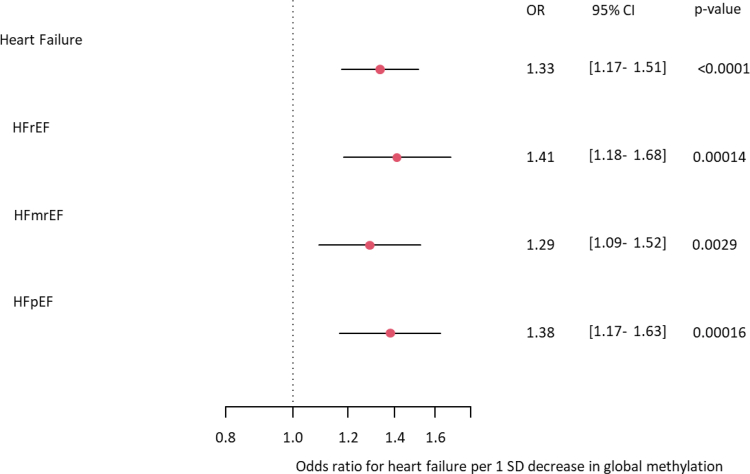
Relation of the decrease in global methylation with the presence of HF. OR—odds ratio. CI—confidence interval. HFrEF—HF with reduced ejection fraction. HFmrEF—HF with mildly reduced ejection fraction. HFpEF—HF with preserved ejection fraction. OR, CI and p value generated using logistic regression with robust estimate covariance. Models adjusted for age and sex.

**Fig. 4:**
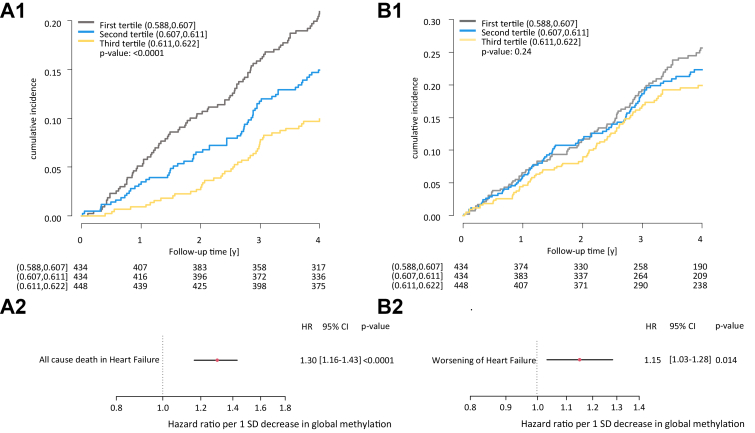
Clinical outcome and global methylation in HF. A 1—Cumulative incidence of all-cause death by tertiles of global methylation. B 1—Cumulative incidence of worsening of HF by tertiles of global methylation. A 2—Risk of all-cause death per one SD decrease of global methylation. B 2—Risk of worsening of HF per one SD decrease of global methylation. HR, CI and p value generated using Cox regression. HR—hazard ratio. CI—confidence interval. Models adjusted for age and sex.

Global methylation was associated with all three heart failure phenotypes (Fig. 3). The strongest association was observed with HFrEF (Odds Ratio, OR: 1.41 [95%, CI: 1.18–1.68]), while the weakest association was with HFmrEF (OR: 1.29 [95% CI: 1.09–1.52]). The association with HFpEF fell in between (OR: 1.38, 95% CI: 1.17–1.63). A higher all-cause death (Fig. 4A1) was observed in individuals with lower global methylation (Hazard Ratio per SD of global methylation = 1.30 [95% CI:1.16–1.43], p = 2.9 × 10–6; Fig. 4A2). This effect was independent of age as demonstrated in a subgroup analysis with age matching between tertiles (Supplemental Fig. S9A and B) and persisted with adjustment for cardiovascular risk factors (Supplemental Fig. S10A). Worsening of HF was associated with global methylation (HR per SD of global methylation = 1.15 [1.03–1.28], p = 0.014; Fig. 4B1 and B2), effect was attenuated after adjustment for cardiovascular risk factors (Supplemental Fig. S10B).

### Chromosomal and regional variability of methylation

**Fig. 5:**
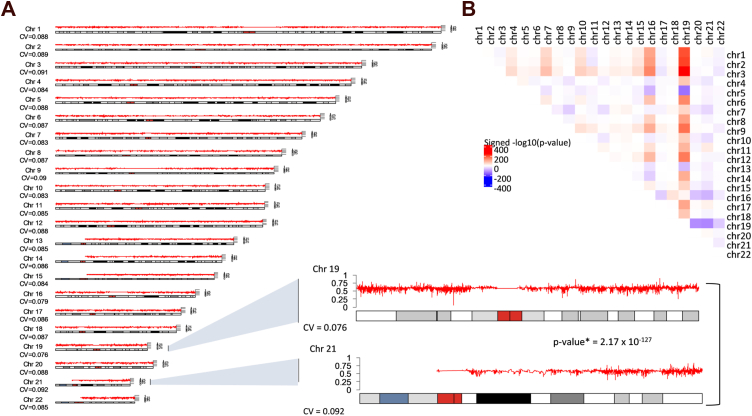
Interchromosomal difference in heart failure related methylation. A—Variability of DNA methylation in HF across autosomes. B—Strength of difference of CV scores between autosomes. ∗p-values generated using asymptotic test for the equality of coefficients of variation. Direction of the strength of p-value refers to the comparison of columns vs. rows, e.g. chr21 vs. chr19 is negative, meaning chr19 is less variable.

When investigating the chromosomal variability of methylation, chromosome 21 had the highest coefficient of variation for methylation (CV = 0.092), and chromosome 19 the lowest (CV = 0.076; Fig. 5A, p for difference = 2.17 × 10^−127^). Chromosome 19 exhibited a high divergence value (d-value, a measure of how far each sample's CV deviates from the estimated population CV. Notably, chromosomes 1–3, and 9 also showed high d-values (p = 4.12 × 10^−205^, 1.53 × 10^−216^, 6.91 × 10^−244^, 7.81 × 10^−173^ respectively; Fig. 5B).

**Fig. 6:**
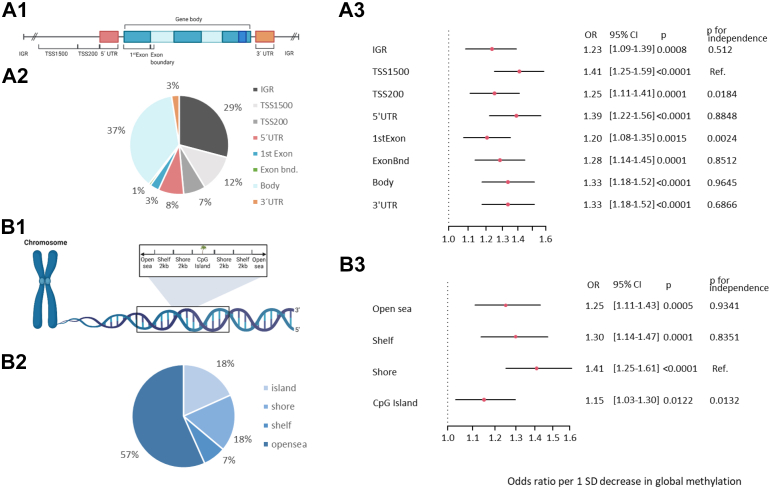
Regional demethylation in heart failure. A 1.—Diagram of gene regions. A 2.—Distribution of gene regions on a methylation array. A 3.—Demethylation in gene regions associated with presence of HF. B 1.—Diagram of genome regions in relation to CpG islands. B 2.—Distribution of genome regions on a methylation array. B 3.—Demethylation in genome regions associated with presence of HF. Models adjusted for age and sex. TSS—transcription start site. UTR—untranslated region. IGR—intergenic region. KB—kilobase (1000 bases). OR—odds ratio. CI—confidence interval. Ref—reference. ORs, CIs and p-values were generated with robust logistic regression. p-values for independence were generated with robust logistic regression by additionally adjusting for reference region. Parts of the figure created in https://BioRender.com.

Among gene regions (average methylation for specified gene region across the whole genome; Fig. 6A1 and A2), the highest association of global methylation with the presence of HF was observed in TSS1500 (OR = 1.41 [1.25–1.59], p = 7.9 × 10–8) and in the 5′ UTR (OR = 1.39 [1.22–1.56], p = 2.7 × 10–7; Fig. 6A3). In the comparative analysis of genomic regions in relation to CpG islands (Fig. 6B1 and B2), methylation in the flanking shore regions was most strongly associated with the presence of HF (OR = 1.41 [1.25–1.61], p = 3.4 × 10–8), while methylation within CpG islands themselves showed weaker association (OR = 1.15 [1.03–1.30], p = 0.0122; Fig. 6B3). Adjustment for the region with highest effect size (TSS1500 for gene regions and shore for CpG island-related regions) resulted in the effect of 5'UTR becoming not relevantly associated anymore, while the association of methylation in CpG islands remained unchanged. All associations remained after false discovery rate correction (Supplemental Table S2). Methylation in CpG islands interacted with all three other regions, i.e., the open sea, shelf and shore region (p = 3.88 × 10^−6^, 1.43 × 10^−6^, 0.0023 respectively), positively contributing to the probability of present HF, while these three regions themselves did not interact with each other (Table 2).

**Table 2:** Interaction of methylation in genome regions.

	Estimate	95% CI	p-value
CpG Island			
Island x Open sea	0.19	0.11; −0.27	3.88 × 10^−6^
Island x Shelf	0.21	0.12; −0.29	1.43 × 10^−6^
Island x Shore	0.17	0.05; −0.26	0.0023
Shelf x Open sea	0.03	−0.05; −0.11	0.521
Shore x Open sea	0.04	−0.05; −0.15	0.331
Shore x Shelf	0.08	−0.01; −0.18	0.08

Interaction performed using individual regression models with the interaction term as dependent variable, with each row representing a separate model (for the first row the model equation was HF status ∼ Island methylation ∗ Open sea methylation). Estimate indicates log Odds ratio of the interaction term.

## Discussion

The main findings show that, on a global scale, individuals with HF exhibit lower levels of DNA methylation compared to individuals without HF. Importantly, the difference in methylation was not driven by chronological age discrepancies between the groups, since it was determined by estimating the age at which individuals without HF have the same global methylation levels observed in the HF group. On average, the global methylation profile of a person with HF resembled that of a person without HF who is nearly 19 years older. Additionally, this study revealed that premature ageing, as indicated by epigenetic clocks, correlated with all-cause death and a worsening of HF. Cardiovascular risk factors apart from age and sex, were not independently associated with global methylation. There was no substantial difference between HF phenotypes at the level of global methylation.

Given the substantial baseline age difference between HF cases and controls, additional resampling and matching analyses were performed to address potential residual confounding. The persistence of the methylation differences across 1000 age-matched iterations supports that the observed associations are unlikely to be driven solely by age imbalance or cohort structure. These findings strengthen confidence in the robustness of the results.

While the absolute difference in global methylation was small (0.2%), such marginal shifts across a highly conserved genome can reflect cumulative changes at millions of sites. The prognostic value of this signature is underscored by its ability to predict mortality, suggesting its utility as a systemic marker rather than a direct mechanistic driver.

When examining global methylation at the level of individual chromosomes, methylation patterns varied across autosomes between HF and non-HF individuals. Chromosome 21 displayed the highest variability in methylation, while chromosome 19 exhibited the lowest. Interestingly, chromosome 19 also differed most in its variation compared to the majority of other chromosomes.

Another noteworthy finding was that gene and genome regions were differentially methylated between individuals with and without HF, but CpG islands were not. However, CpG island methylation appeared to regulate the strength of the relationship between HF and methylation in other gene regions.

Most studies that have examined changes in global methylation in cardiovascular disease have reported an increase in global methylation associated with disease state.^22^ However, these findings are largely derived from non-microarray-based methods, such as ELISA, radiolabeling, or methylation-specific PCR, which quantify total genomic methyl-cytosine content rather than specific CpG sites. In contrast, the present findings of global hypomethylation were obtained using high-resolution arrays. This discrepancy may be methodological, as microarrays provide a representative average across hundreds of thousands of specific genomic regions. Present results are consistent with recent large-scale evidence^23^ suggesting that global hypomethylation is a hallmark of biological ageing and chronic metabolic stress. While one early radiolabeling study of 17 cases previously reported hypomethylation in atherosclerosis,^24^ the present larger-scale analysis suggests that systemic hypomethylation may be a more prevalent feature of HF than previously recognised using less granular quantification methods.

The difference in DNA methylation between HF and control individuals fits with the previously demonstrated pattern, where higher epigenetic age compared to calendar age indicates a poorer health.^25^ This difference was further highlighted by the correlation between premature ageing, derived from GrimAge and Hannum Clock, with worsening of HF and with overall mortality. This shows that not only are patients with heart failure epigenetically older, which is a known sign of deteriorating health condition,^26^ but that the presence and severity of HF also accelerates ageing itself. It is important to note that GrimAge correlated more strongly with all tested conditions (i.e., severity, worsening and mortality) when compared to Hannum Clock. This indicates that GrimAge, which was trained to predict lifespan and healthspan, captures the signature of HF and HF-related changes more accurately than the Hannum Clock, which was trained to predict chronological age only.

Relevant differences between associations of global methylation and specific phenotypes of HF were not observed in this cohort. This may be due to substantial differences in terms of which specific CpG sites were methylated (dependent on different etiologies of the phenotypes), rather than extent of methylation across the genome. Such site-specific variations may align with findings from previous research highlighting analogous differences at the metabolomic^27^ as well as genetic^28^ level.

Chromosome (Chr) 19 stood out with regard to regional variability of methylation among all others in the current study. This chromosome exhibits an unusual DNA composition such as the highest GC content of any chromosome, especially outside gene clusters,^29^ which relates to DNA methylation. There is evidence that some QTLs on Chr 19 may be tied to LDL cholesterol concentration,^30^ which would also directly link them to CVD. Chr 16 also exhibited similar characteristics to Chr 19. It is the Chr with the second least variability of methylation, and has also shown increased differences in its CV when compared to the other chromosomes. There is evidence suggesting that some regions of Chr 16 could be involved in vascular calcification in diabetes mellitus in the setting of HF.^31^

Other noteworthy findings of this study relate to regional differences in methylation between individuals with and without HF. Many prior studies have focused on CpG islands as regions where differential methylation is most informative for discriminating pathological conditions.^32^ However, the present study showed that methylation in CpG islands showed weaker discrimination for HF vs. non-HF, and that the most discriminatory regions were those immediately adjacent to the CpG islands, i.e., the “shore regions”. Shore regions play a role in breast cancer,^33^ but there is little to no evidence linking them to HF, with only one study linking them to acute coronary syndrome.^34^

Only methylation in the CpG islands was found to interact with all 3 other genome regions, whereas they themselves have not shown any interaction with each other. This suggests a potential modulatory role for CpG islands, where they may serve as a regulatory point influencing the methylation of neighbouring regions in the context of HF. Moreover, after adjusting for the region with the strongest association with the presence of heart failure (shore in case of genome and TSS1500 in case of the gene regions) the effect sizes of most other regions were substantially reduced, such that only the CpG island, first exon and TSS200 remained associated with the presence of HF. While this suggests that these regions might contribute to HF risk through pathways distinct from other regions,^35^ these findings should be considered hypothesis-generating. Further mechanistic studies are required to define the precise regulatory architecture underlying these associations.

While previous studies have primarily examined epigenetic age acceleration^36^ or locus-specific CpG associations in heart failure, these approaches capture only a limited fraction of the methylome. In contrast, this work investigated whether heart failure is accompanied by system-wide alterations in the magnitude and genomic distribution of DNA methylation, thereby providing insight into the broader epigenomic or, more specifically, methylome-based architecture of heart failure beyond established epigenetic clock measures.

The clinical relevance of global methylation may lay in their ability to capture the cumulative systemic impact of HF, extending beyond the acute haemodynamic information provided by markers such as NT-proBNP. These broad structural changes to the methylome are reminiscent of the utility of Polygenic Risk Scores in modern genetics, where the aggregation of thousands of small genomic variations provides a robust measure of disease susceptibility and progression that individual markers cannot capture.^37^ In the present study, global hypomethylation and accelerated epigenetic ageing serve as a molecular ‘summary’ of the environmental effect associated with HF. Establishing these systemic shifts is a necessary foundation for understanding how the HF is encoded at the epigenomic level.

The major strengths of the present study are the large sample size, the comprehensive coverage of the genome provided by the 850 k assay and the granular (sub)clinical information available on each study subject, including long-term follow-up. The study also has limitations: these include the difference in the average age between the two groups, the usage of an array platform, which limits the amount of CpG sites to a predefined set, as opposed to non-targeted approaches such as whole genome sequencing. Furthermore, a limitation of this study is the use of peripheral blood DNA methylation, which may not directly reflect localised myocardial epigenetic changes. However, systemic epigenetic signatures can serve as robust proxies for biological ageing and disease state. This is supported by recent work,^38^ which demonstrated that many epigenetic clocks maintain high stability and consistent performance across diverse human and animal tissues. While the present findings primarily reflect circulating epigenetic signatures, they may capture broader systemic changes associated with HF. Additionally, the generalisability of these findings is limited by the composition of the cohorts, which consists primarily of individuals of European descent from Midwest Germany, thus these results may not be representative of all ancestries.

Due to the specific composition of the study cohorts and the original research design, this study was not powered to perform a sex-specific analysis. The divergence in health status between cohorts is a limitation, as the control group was screened to exclude major systemic diseases prevalent in the group with heart failure. While this established a clear epigenetic baseline, it may lead to an overestimation of differences and introduces the risk of residual confounding. Consequently, these results should be interpreted as a comparison between a pathological state and an optimised healthy reference.

The design of the study precludes the establishment of definitive causality, as the observed epigenetic alterations may represent a consequence of HF or its treatment rather than a primary driver. Despite extensive adjustment and repeated age-matching procedures, residual confounding cannot be fully excluded. This is particularly relevant given the case–control design and the use of two independent cohorts, which may differ in comorbidity burden, medication, and overall health status. Therefore, the observed associations should be interpreted with caution and do not imply causality.

In summary, the findings of this study indicate that global and regional DNA methylation patterns are consistently associated with the presence, severity, and clinical outcomes of heart failure, suggesting a system-wide epigenomic shift linked to the disease. Although the absolute differences in global methylation were small, they reflect coordinated changes across a large proportion of the methylome. In addition, epigenetic age acceleration showed robust associations with adverse outcomes, highlighting its potential relevance for risk stratification. These results support global DNA methylation as an integrative marker of biological ageing and disease burden in heart failure. However, given the observational design and use of distinct cohorts, residual confounding cannot be excluded, and causal inference remains limited. Further studies are needed to validate these findings and clarify their clinical utility.

## Contributors

M. Krolevets curated the data, performed the formal analysis, performed investigation, contributed to project administration, contributed to development of the code, interpreted the data, visualised the results, drafted the manuscript, critically revised it, and approved the final version. V. ten. Cate, curated the data, performed investigation, contributed to development of the code, interpreted the data, drafted the manuscript, critically revised it, and approved the final version. J. H. Prochaska contributed to data analysis, interpreted the data, critically revised the manuscript, and approved the final version. A. Schulz performed data analysis, developed software and code, supported visualisation and data interpretation, critically revised the manuscript, and approved the final version. S. Rapp contributed to laboratory data acquisition, supported data interpretation, critically revised the manuscript, and approved the final version. S. Zeid contributed to the conception and design of the revised analysis, interpreted the data, visualised the results, critically revised the manuscript, and approved the final version. S. Tenzer contributed to data acquisition, critically revised the manuscript, supported data interpretation, and approved the final version. M. A. Andrade-Navarro contributed to data acquisition, software, and methodology, critically revised the manuscript, supported data interpretation, and approved the final version. A. Lu contributed to methodology and software, critically revised the manuscript, supported data interpretation, and approved the final version. K. Strauch contributed to data acquisition, critically revised the manuscript, supported data interpretation, and approved the final version. A. K. Schuster contributed to data acquisition, critically revised the manuscript, supported data interpretation, and approved the final version. M. E. Beutel contributed to data acquisition, critically revised the manuscript, supported data interpretation, and approved the final version. I. Heinrich contributed to data acquisition, critically revised the manuscript, supported data interpretation, and approved the final version. J. Weinmann-Menke contributed to data acquisition, critically revised the manuscript, supported data interpretation, and approved the final version. K. J. Lackner contributed to data acquisition, critically revised the manuscript, supported data interpretation, and approved the final version. P. Lurz contributed to data acquisition, funding acquisition, critically revised the manuscript, supported data interpretation, and approved the final version. S. Horvath contributed to formal analysis, investigation, methodology, software, as well as provided supervision and critically revised the manuscript, supported data interpretation, and approved the final version. C. Niehrs contributed to data and funding acquisition, methodology, formal analysis, supervision as well as critically revised the manuscript, supported data interpretation, and approved the final version. P. S. Wild, contributed to the data curation, contributed to formal analysis, acquired funding, contributed to methodology, project administration and supervised the work, as well as interpreted the data, contributed to visualisation, drafted the manuscript, critically revised the manuscript, and approved the final version. All authors conceived and designed the study. All authors have reviewed the manuscript for intellectual content and approved its submission. M. Krolevets, V. ten. Cate, A. Schulz and P.S. Wild have accessed and verified the data.

## Data sharing statement

The data that support the findings of this study are not openly available due to reasons of sensitivity and human data privacy protection policy. The data may be shared on reasonable request to the corresponding author. Data are located in controlled access data storage at University Medical Centre Mainz.

## Declaration of interests

The Regents of the University of California are the sole owner of patents and patent applications directed at epigenetic biomarkers for which Ake Tzu-Hui Lu and Steve Horvath is a named inventor; Steve Horvath is a founder and paid consultant of the non-profit Epigenetic Clock Development Foundation that licences these patents. Christof Niehrs reports Research grants on Wnt signalling, and nucleic acid modifications from DFG (Deutsche Forschungsgemeinschaft), state of Baden Württemberg and Rhineland Palatinate. Outside the submitted work, Philipp Wild reports grants from Bayer AG, non-financial grants from Philips Medical Systems, grants and consulting fees from Boehringer Ingelheim, grants and consulting fees from Novartis Pharma, grants and consulting fees from Sanofi-Aventis, grants, consulting and lecturing fees from Bayer Health Care, grants and consulting fees from Daiichi Sankyo Europe, lecturing fees from Pfizer Pharma, lecturing fees from Bristol Myers Squibb, consulting fees from Astra Zeneca, consulting fees and non-financial support from Diasorin and non-financial support from IEM. Stefan Tenzer reports Grant from ReALity (P6 Tenzer funded by the state of Rhineland-Palatinate from 2019 to 2024), curATime grant from Federal Ministry of Education and Research diAMs, FKZ 03ZU1202EA, DIASyM grant from Federal Ministry of Education and Research FKZ 031L0241A/B, FKZ 03LW0241K, German Research Foundation DFG SFB1292/2, TP-Q01. Jürgen Prochaska is a full-time employee of Boehringer Ingelheim International GmbH. Karl Lackner reports consulting fees from Novartis; Abbott Diagnostics. Isabel Heinrich reports Grants from Federal Ministry of Education and Research (BMBF 01EQ2401A). The other authors declare no conflicts of interests.

## Figures and Tables

**Graphical abstract:**
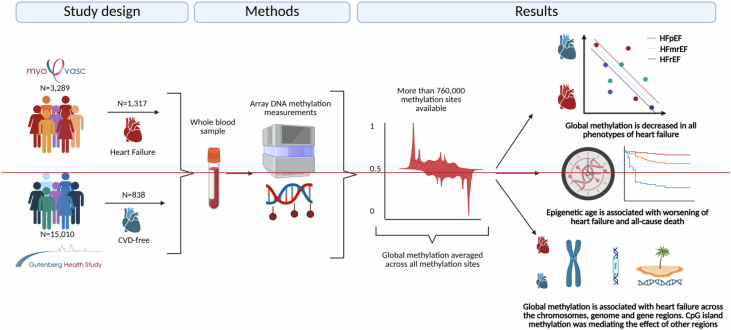
Global and regional methylation is associated with Heart Failure presence and outcome. Legend: Parts of the figure created in https://BioRender.com.
