# Supplementary material for: Global and regional DNA methylation patterns in heart failure: a case–control analysis
Source: eBioMedicine. 2026 Jun 23;129:106340. doi: 10.1016/j.ebiom.2026.106340 (PMC13316289; doi:10.1016/j.ebiom.2026.106340)
Supplement: Supplemental Figs. S1–S10 and Tables S1 and S2 [file mmc1.pdf]

Supplementary Appendix

Global and Regional DNA Methylation Patterns in Heart Failure: A Case–Control Analysis

Table of Contents

Supplementary Figure S1. Assessment of potential batch effects in the methylation data. 3

Supplementary Figure S2. Steps of data normalization and batch correction. 4

Supplementary Figure S3. Assessment of global methylation in the EpiHF cohort. 5

Supplementary Figure S4. Comparison of methylation-inferred cell-type composition in HF cases versus controls. 6

Supplementary Figure S5. Age matched subgroup analysis of heart failure cases and controls. 7

Supplementary Figure S6. Relation of premature ageing with clinical outcome with adjustment for inferred cell-type composition. 8

Supplementary Figure S7. Relation of premature ageing with clinical outcome with adjustment for age, sex, smoking, alcohol consumption and intake of beta blockers and ACE inhibitors. 9

Supplementary Figure S8. Relation of premature ageing with clinical outcome with adjustment for cardiovascular risk factors. 10

Supplementary Figure S9. Incidence of all-cause death in age-matched sample. 11

Supplementary Figure S10. Clinical outcome and global methylation in HF with adjustment for cardiovascular risk factors. 12

Supplementary Table S1. Characteristics of the analysis sample. 13

Supplementary Table S2. FDR-corrected p values for regional methylation analysis. 14

Supplementary Figure S1. Assessment of potential batch effects in the methylation data.

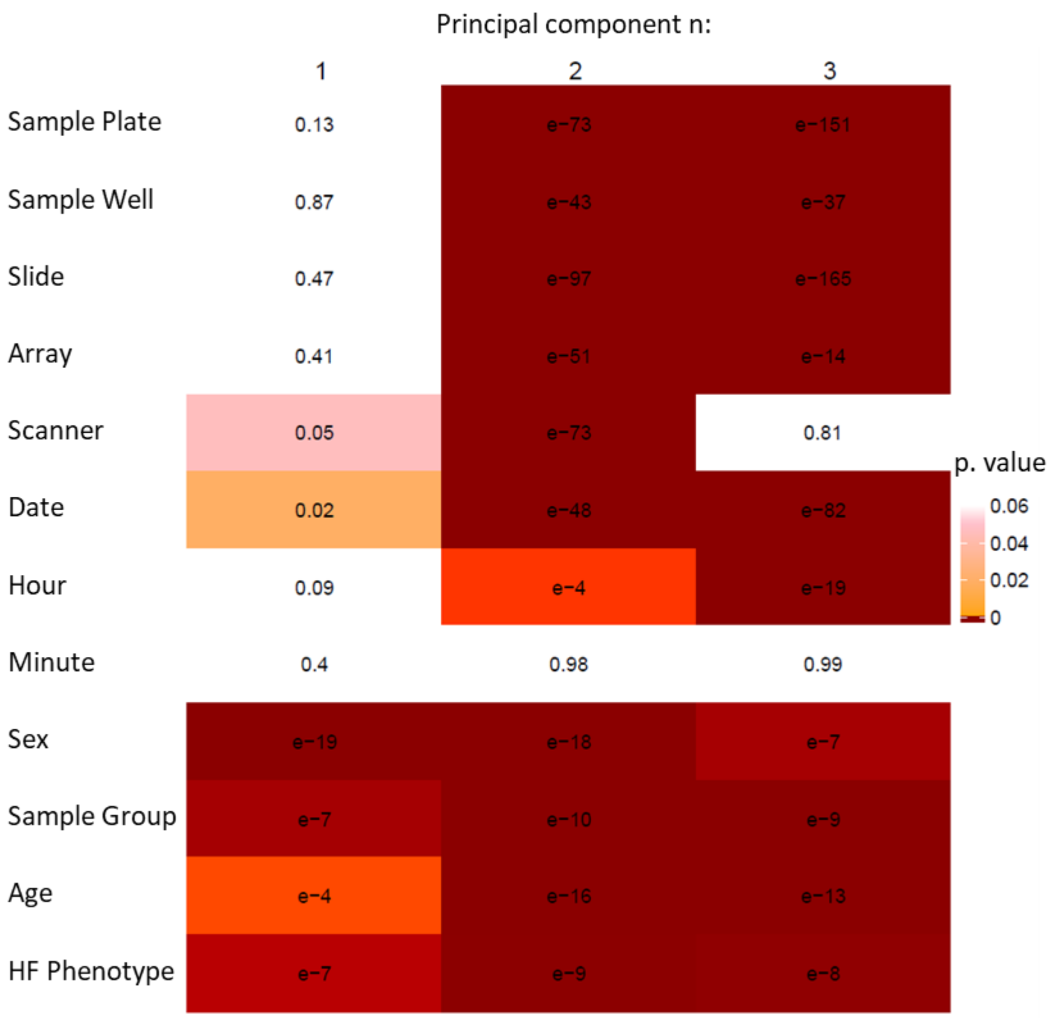

Singular value decomposition of potential batch effects (above) and sample phenotype parameters (below). P value indicates association of the parameters with methylation.

Supplementary Figure S2. Steps of data normalization and batch correction.

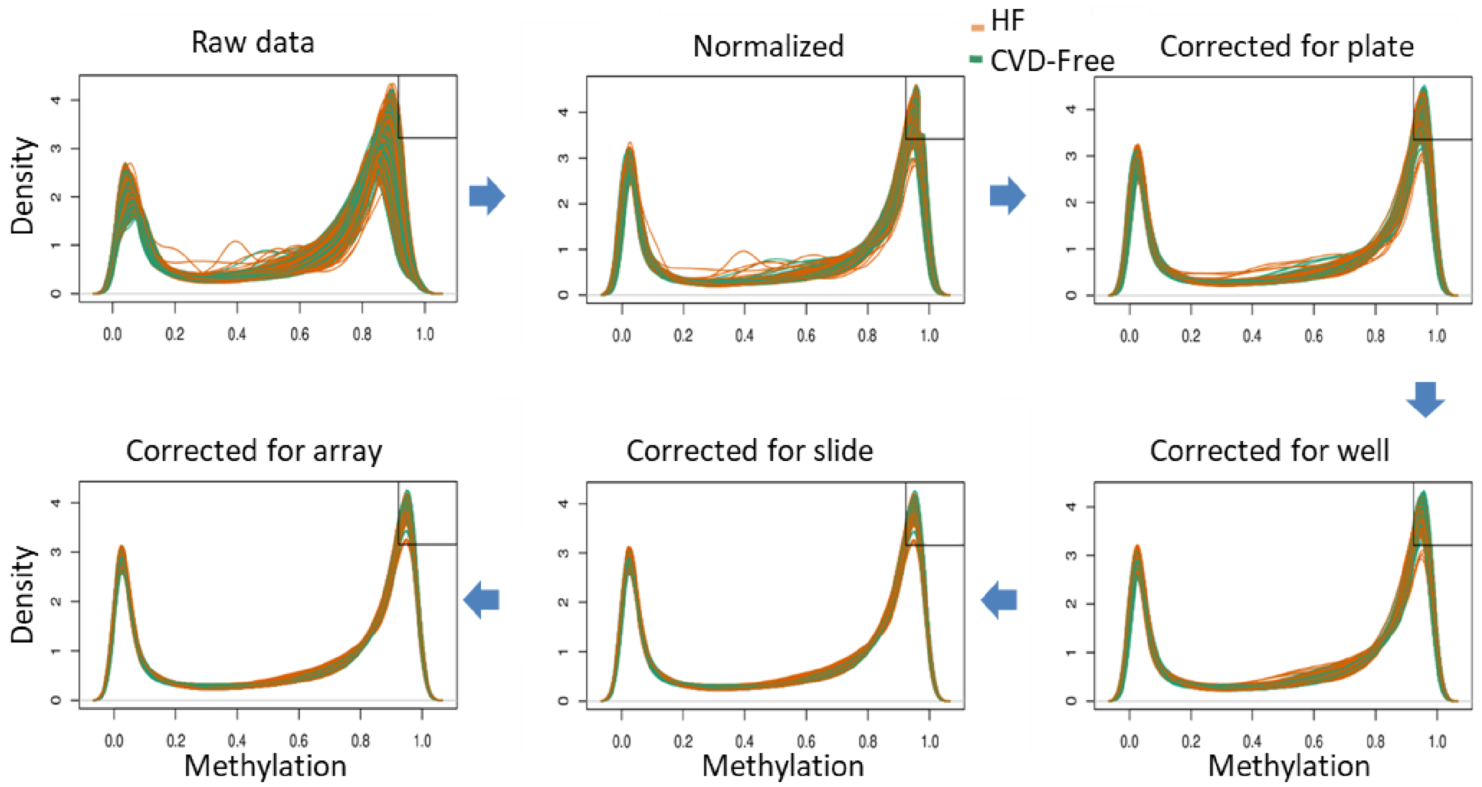

Flow of data pre-processing. Curves indicate average methylation distribution across all CpGs for HF and control groups.

Supplementary Figure S3. Assessment of global methylation in the EpiHF cohort.

A. Comparison of average methylation in the whole HF and control sample.

B. Comparison of average methylation in HF stratified by phenotype.

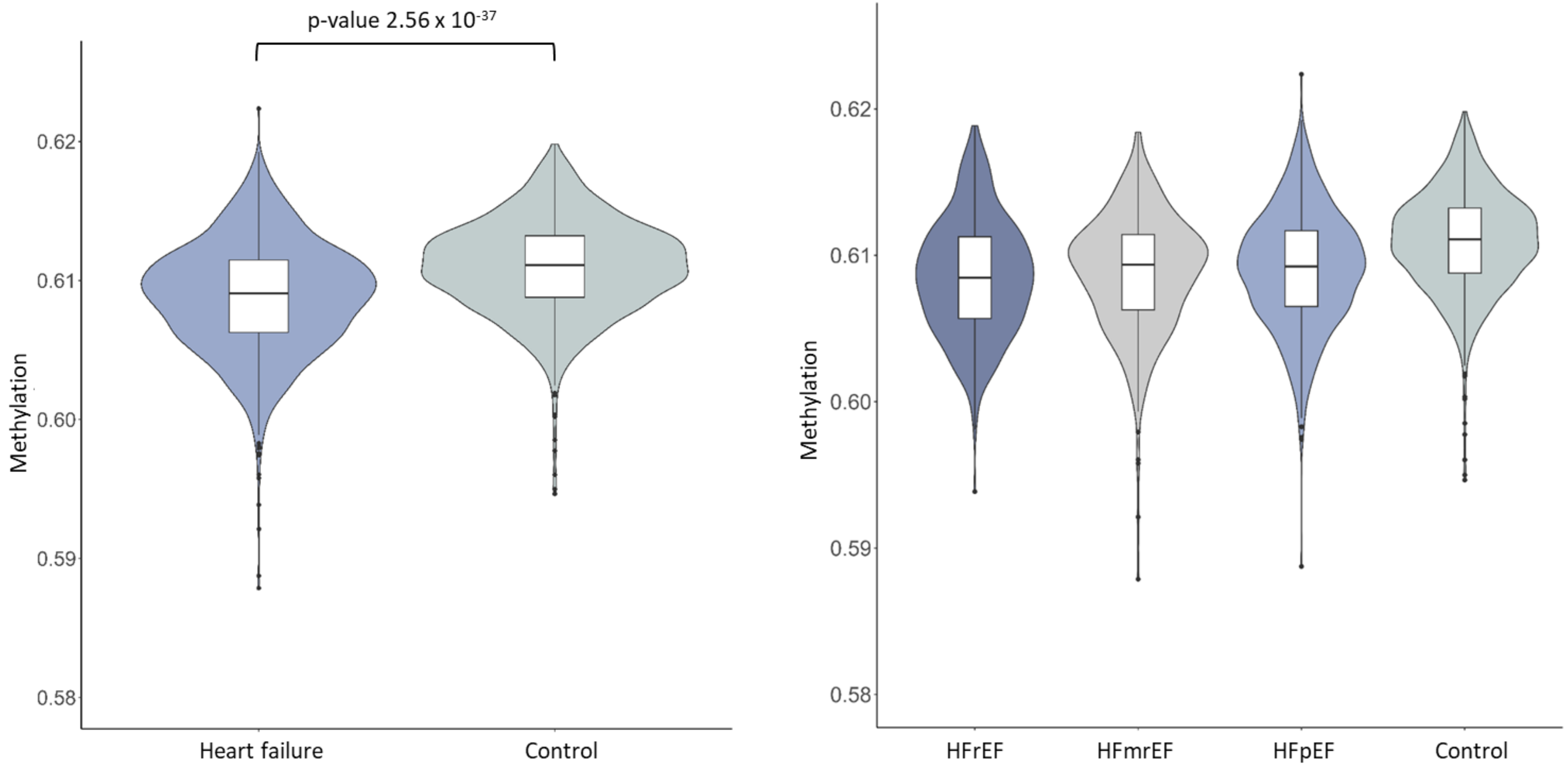

Distribution of methylation in analysis sample (N=2,155). Methylation was defined as percentage of methylated CpG on a given position in Genome I.e. 0 = 100% are demethylated, 1 = 100% are methylated

Supplementary Figure S4. Comparison of methylation-inferred cell-type composition in HF cases versus controls.

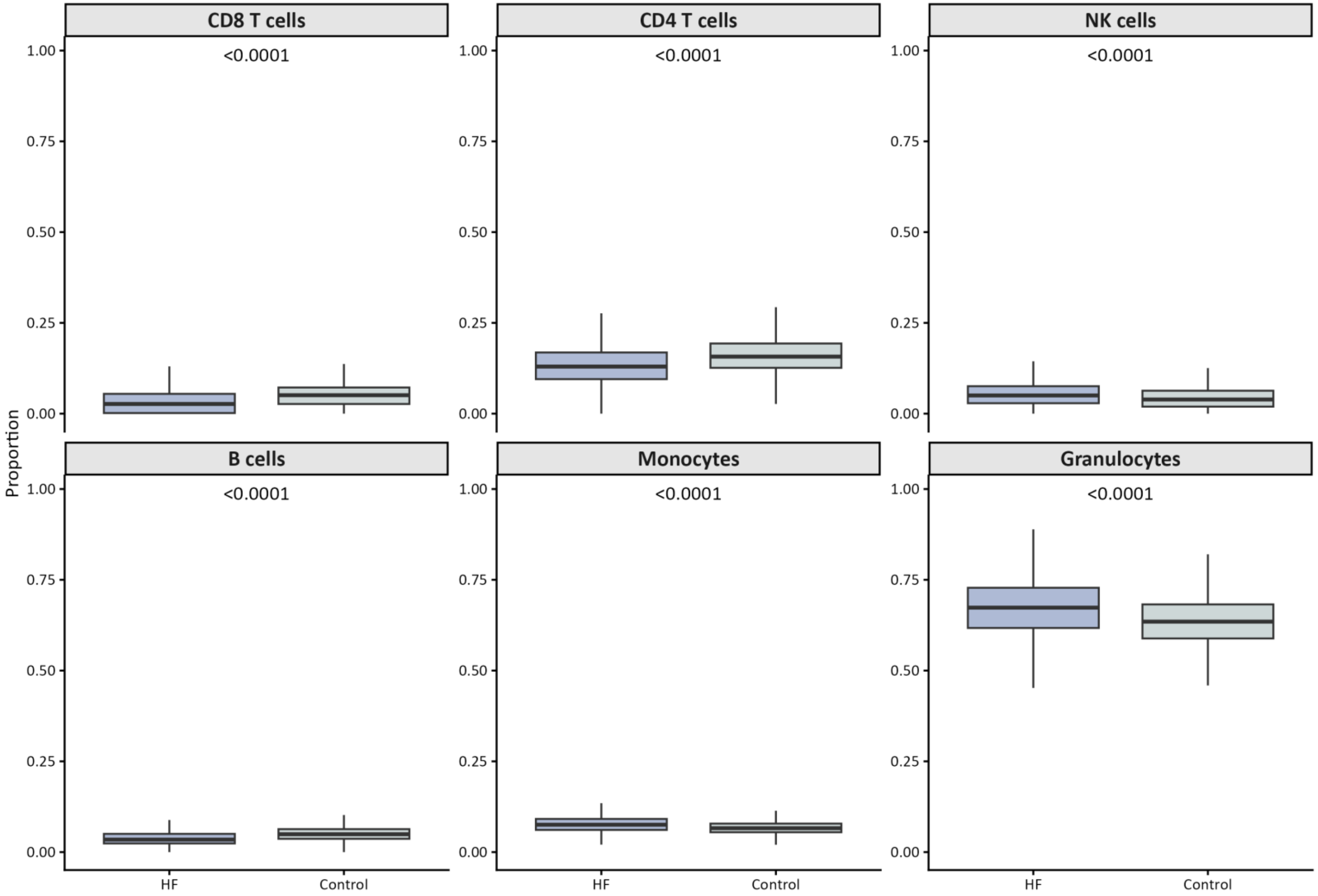

Supplementary Figure S5. Age matched subgroup analysis of heart failure cases and controls.

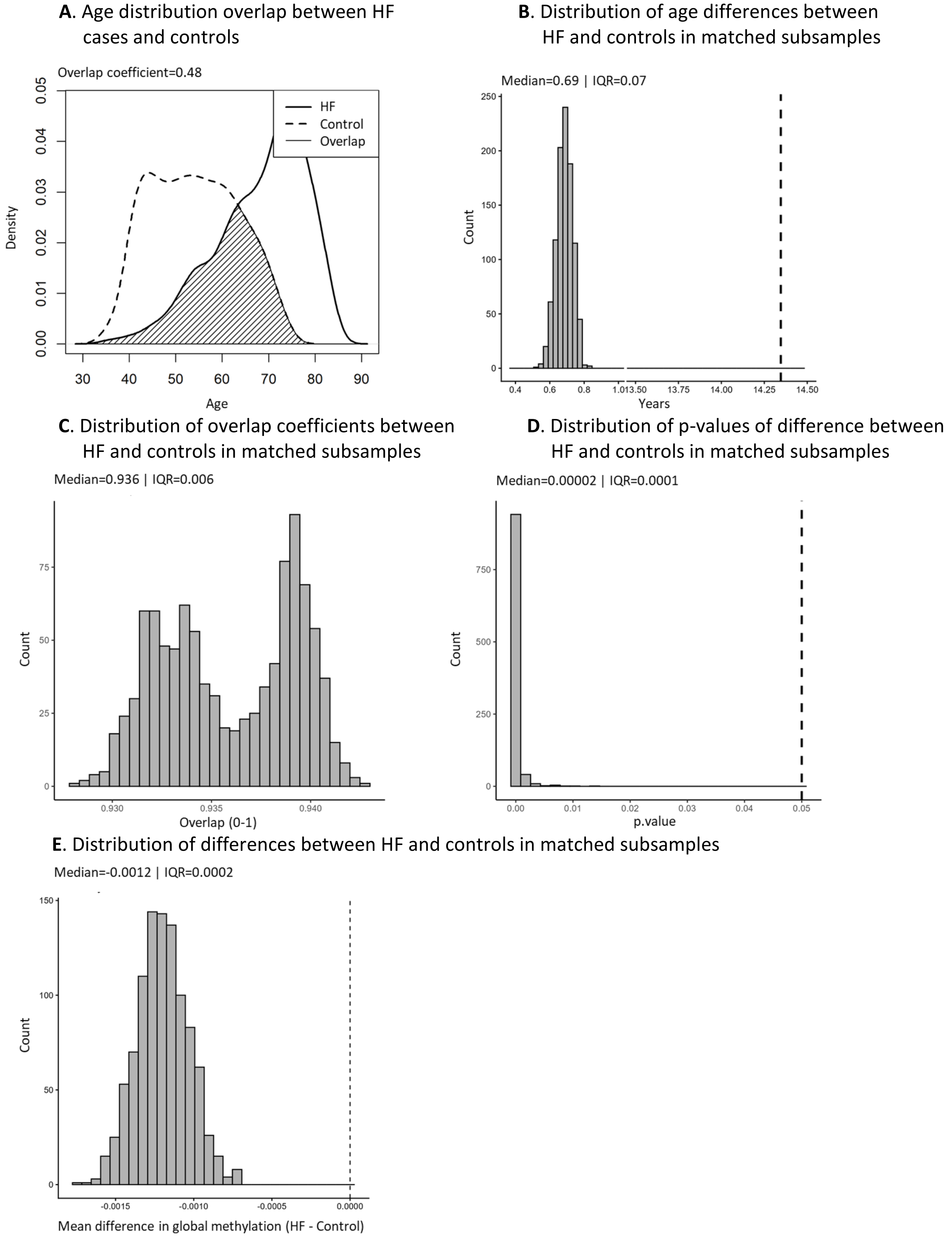

Panel A. Age distribution overlap between HF cases and controls. Panel B. Distribution of age differences between HF and controls in matched subsamples. Panel C. Distribution of overlap coefficients between HF and controls in matched subsamples. Panel D. Distribution of p-values of difference between HF and controls in matched subsamples. Panel E. Distribution of differences between HF and controls in matched subsamples. HF – heart failure; IQR – inter quartile range.

Supplementary Figure S6. Relation of premature ageing with clinical outcome with adjustment for inferred cell-type composition.

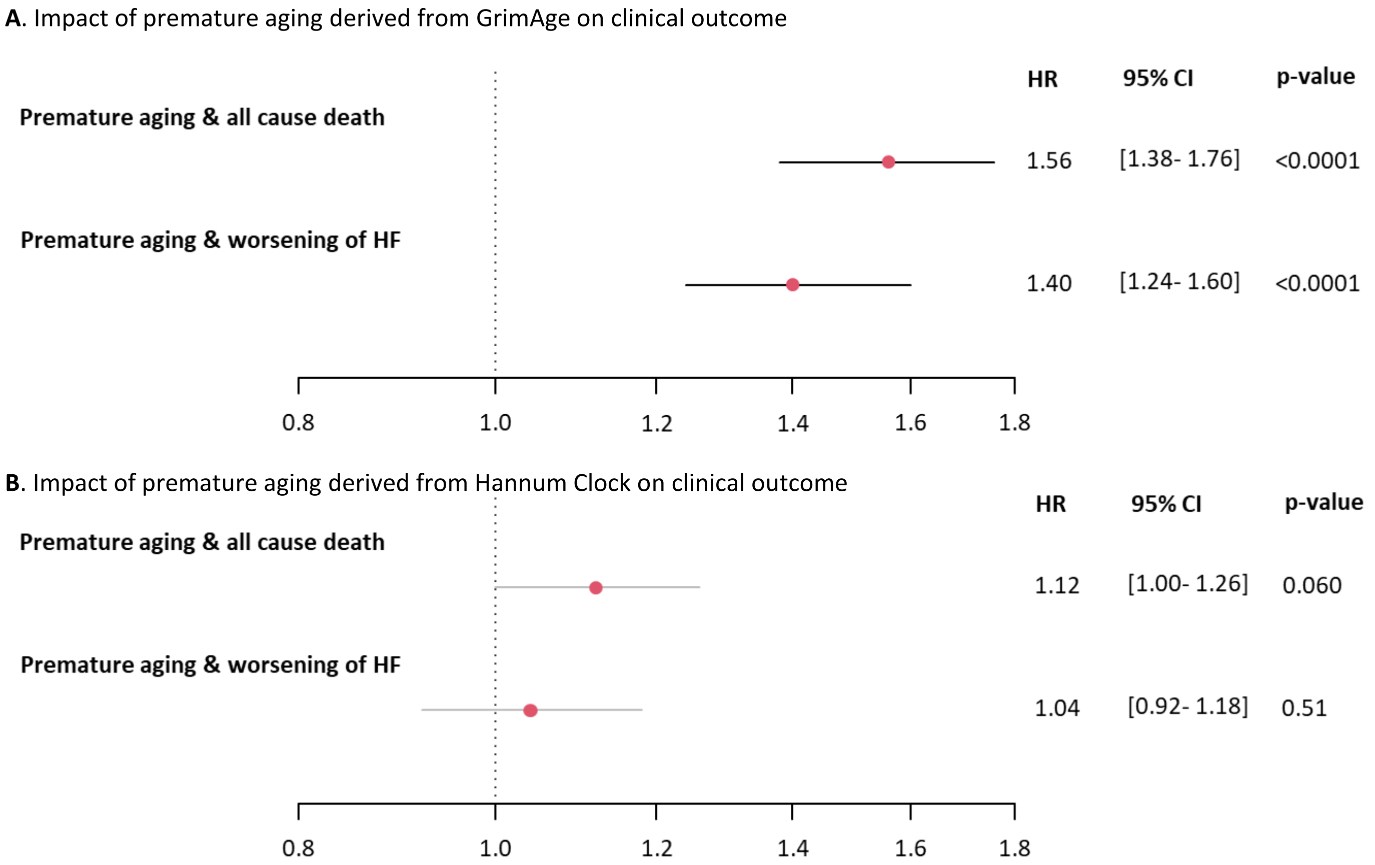

Premature aging – residuals from a linear regression of calendar age predicting epigenetic age. The residuals represent the portion of epigenetic age not explained by calendar age, where positive residuals indicate accelerated aging with respect to one's calendar age. Premature ageing was derived from Hannum Clock and GrimAge. Accelerated aging and worsening of HF generated using Cox regression. HR – hazard ratio. CI – confidence interval. Models adjusted for age, sex as well as composition of CD8+ T-cells, CD4+ T-cells, Natural Killer cells, B-cells, Monocytes, and Granulocytes inferred from methylation.

Supplementary Figure S7. Relation of premature ageing with clinical outcome with adjustment for age, sex, smoking, alcohol consumption and intake of beta blockers and ACE inhibitors.

A. Impact of premature aging derived from GrimAge on clinical outcome

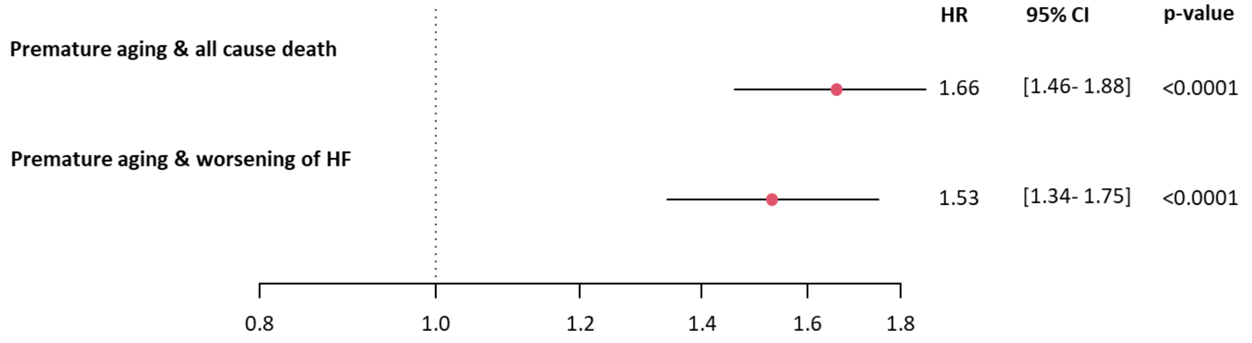

B. Impact of premature aging derived from Hannum Clock on clinical outcome

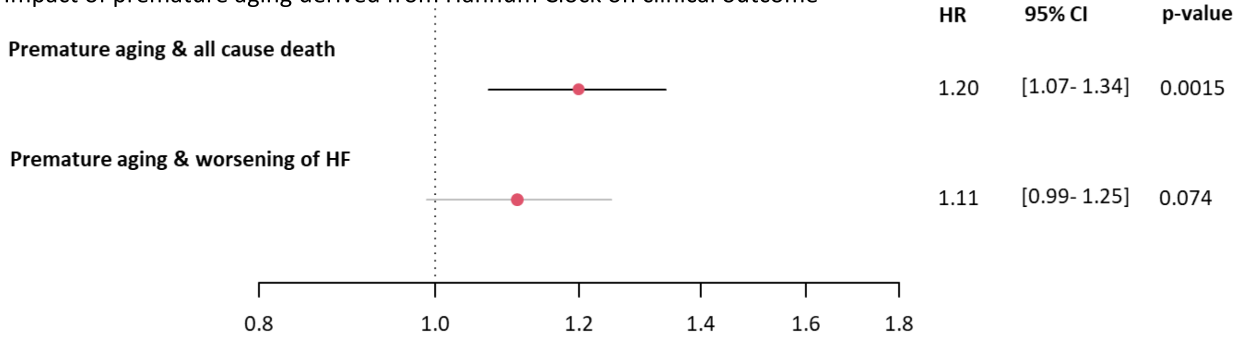

Premature aging – residuals from a linear regression of calendar age predicting epigenetic age. The residuals represent the portion of epigenetic age not explained by calendar age, where positive residuals indicate accelerated aging with respect to one's calendar age. Premature ageing was derived from Hannum Clock and GrimAge. Accelerated aging and worsening of HF generated using Cox regression. HR – hazard ratio. CI – confidence interval. Models adjusted for age, sex smoking status, intake of beta blockers and ACE inhibitors.

Supplementary Figure S8. Relation of premature ageing with clinical outcome with adjustment for cardiovascular risk factors.

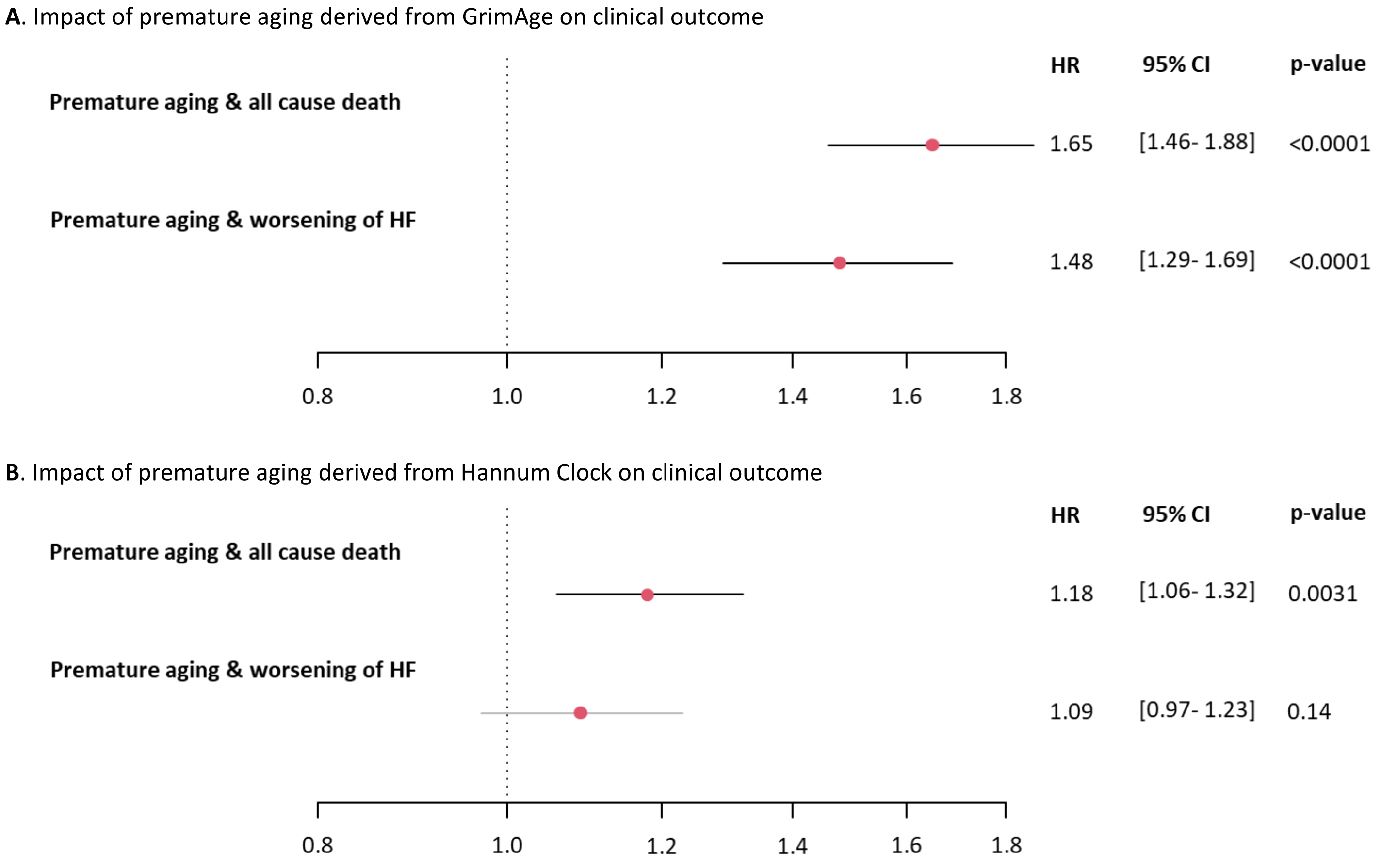

Premature aging – residuals from a linear regression of calendar age predicting epigenetic age. The residuals represent the portion of epigenetic age not explained by calendar age, where positive residuals indicate accelerated aging with respect to one's calendar age. Premature ageing was derived from Hannum Clock and GrimAge. Accelerated aging and worsening of HF generated using Cox regression. HR – hazard ratio. CI – confidence interval. Models adjusted for age, sex diabetes type 2 mellitus, obesity, smoking status, arterial hypertension, dyslipidemia and family history of myocardial infarction or stroke.

Supplementary Figure S9. Incidence of all-cause death in age-matched sample.

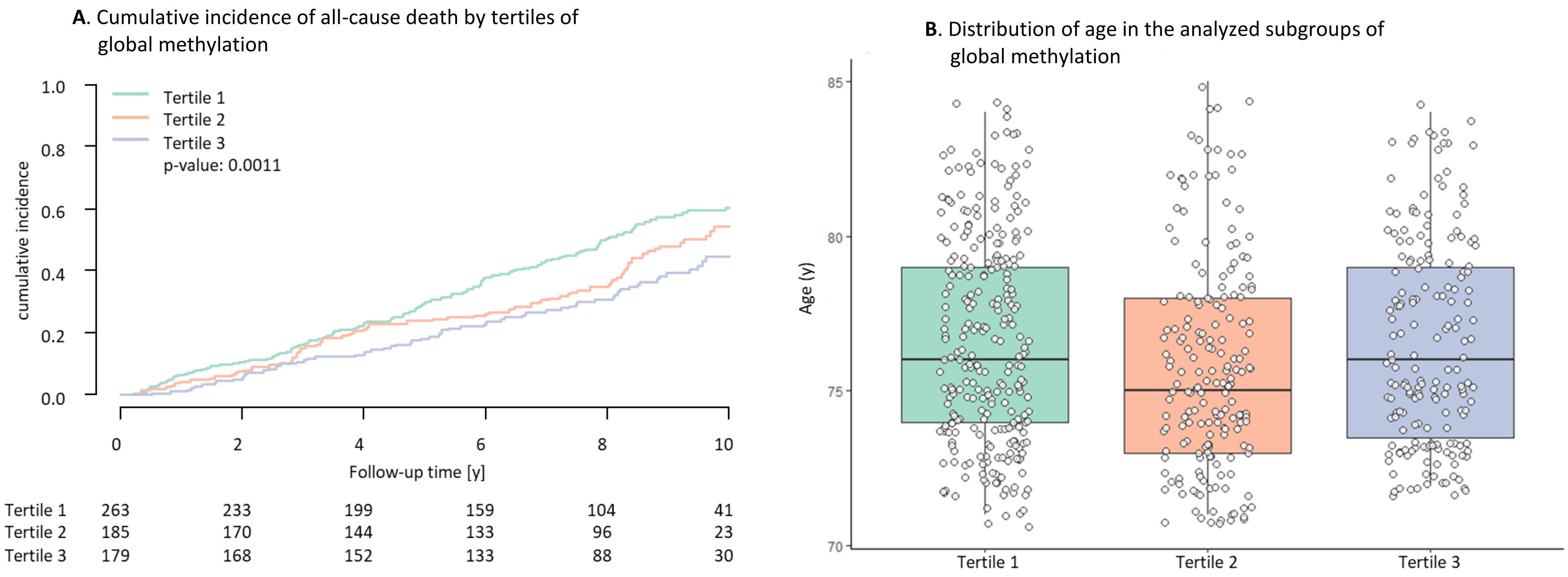

Supplementary Figure S10. Clinical outcome and global methylation in HF with adjustment for cardiovascular risk factors.

A. Risk of all-cause death per one SD decrease of global methylation

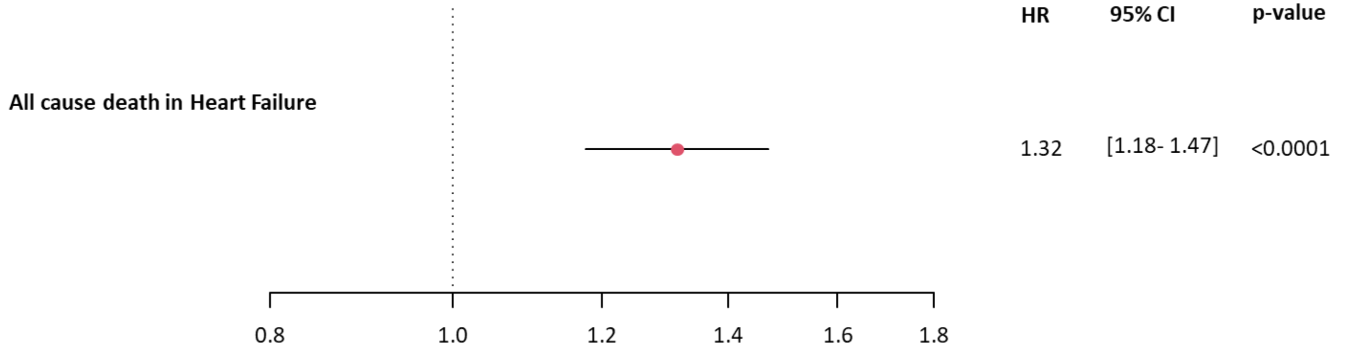

B. Risk of worsening of HF per one SD decrease of global methylation

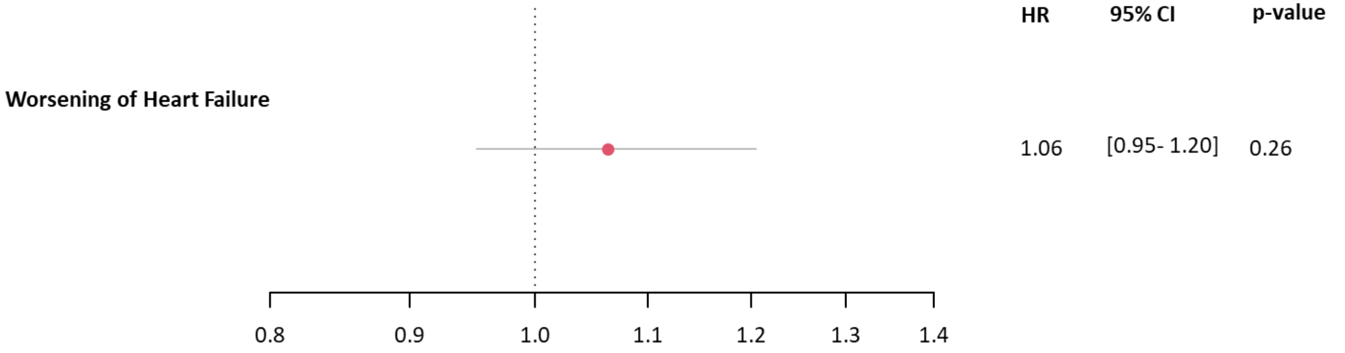

Hazard ratio of specified outcome is generated using Cox regression. HR – hazard ratio. CI – confidence interval. Models adjusted for age, sex diabetes type 2 mellitus, obesity, smoking status, arterial hypertension, dyslipidemia and family history of myocardial infarction or stroke.

Supplementary Table S1. Characteristics of the analysis sample.

Parameter	Individuals with clinically established heart failure	Individuals w/o clinically established heart failure and CVD
N	1,317	838
Age [y]	71·0 (62·0/76·0)	54·0 (46·0/61·1)
Female sex	32·0% (421)	49·0% (411)
NT-proBNP [pg/mL]	461·5 (197·0/1183·8)	44·62 (21·63/74·71)
HF phenotypes		
HFrEF	15·3% (329)	0% (0)
HFmrEF	17·6% (383)	0% (0)
HFpEF	28·1% (605)	0% (0)
Cardiovascular risk factors		
Arterial hypertension	81·9% (1079)	46·4% (389)
Diabetes mellitus	31·4% (414)	5·0% (42)
Dyslipidemia	80·7% (1063)	30% (251)
Family history of MI/Stroke	24·8% (327)	21·5% (180)
Obesity	38·7% (510)	21·2% (178)
Smoking	14·0% (184)	17·4% (146)
Comorbidities		
Atrial fibrillation	37·3% (491)	0% (0)
Chronic kidney disease*	24·0% (316)	0·1% (1)
COPD	15·9% (209)	0% (0)
Coronary artery disease	51·9% (684)	0% (0)
Peripheral artery disease	10·8% (142)	0% (0)
Hx Cancer	19·4% (256)	0% (0)
Hx Myocardial infarction	33·1% (436)	0% (0)
Hx Stroke	11·6% (153)	0% (0)
Hx Venous thromboembolism	11·2% (148)	0% (0)

* Chronic kidney disease defined as estimated glomerular filtration rate (eGFR) < 60 ml/min/1.73m^2. S/P – status post; [y] – years; COPD - chronic obstructive pulmonary disease; HFpEF - heart failure with preserved ejection fraction; HFrEF -heart failure with reduced ejection fraction; HFmrEF - heart failure with mildly reduced ejection fraction; NT-proBNP - B-type natriuretic peptide; CVD – cardiovascular desiase; MI – myocardial infarction

Supplementary Table S2. FDR-corrected p values for regional methylation analysis.

Genome region	Base	Independent	Gene region	Base	Independent
Open Sea	0·0006	0·9341	IGR	0·0009	0·9645
Shelf	0·0001	0·9341	TSS1500	<0·0001	0·9645
Shore	<0·0001	<0·0001	TSS200	0·0002	0·0738
CpG Island	0·0122	0·0263	5´UTR	<0·0001	0·9645
			1stExon	0·0015	0·0192
			ExonBnd	0·0002	0·9645
			Body	<0·0001	0·9645
			3´UTR	<0·0001	0·9645

False discovery rate-corrected p values of the regional methylation analysis. Base refers to p values of association between regional methylation and HF status, Independent refers to the same analysis adjusted for the effect of the strongest region (Shore for genome region and TSS1500 for gene region)
